# Identification of genetic variants controlling diosgenin content in *Dioscorea zingiberensis* tuber by genome-wide association study

**DOI:** 10.1186/s12870-024-05133-1

**Published:** 2024-06-13

**Authors:** Shi xian Sun, Yanmei Li, Lu Jia, Shili Ye, Yunpeng Luan

**Affiliations:** 1 Yunnan Key Laboratory of Plateau Wetland Conservation, Restoration and Ecological Services, Southwest Forestry University, Kunming, 650224 China; 2 Department of Life Technology Teaching and Research, School of Life Science, Southwest Forestry University, Kunming, 650224 China; 3 Faculty of Mathematics and Physics, Southwest Forestry University, Kunming, 650224 China; 4 The First Affiliated Hospital of Yunnan University of Traditional Chinese Medicine, Kunming, 650021 China; 5 Key Laboratory for Forest Resources Conservation and Utilization in the Southwest Mountains of China, Ministry of Education, Southwest Forestry University, Kunming, 650021 China

**Keywords:** *Dioscorea Zingiberensis*, Diosgenin, Breeding, P450, Enzyme

## Abstract

**Background:**

Diosgenin is an important steroidal precursor renowned for its diverse medicinal uses. It is predominantly sourced from *Dioscorea* species, particularly *Dioscorea zingiberensis*. *Dioscorea zingiberensis* has an ability to accumulate 2–16% diosgenin in its rhizomes. In this study, a diverse population of 180 *D. zingiberensis* accessions was used to evaluate the genomic regions associated with diosgenin biosynthesis by the genome wide association study approach (GWAS).

**Results:**

The whole population was characterized for diosgenin contents from tubers by gas chromatography mass spectrometry. The individuals were genotyped by the genotyping-by-sequencing approach and 10,000 high-quality SNP markers were extracted for the GWAS. The highest significant marker-trait-association was observed as an SNP transversion (G to T) on chromosome 10, with 64% phenotypic variance explained. The SNP was located in the promoter region of *CYP94D144* which is a member of P450 gene family involved in the independent biosynthesis of diosgenin from cholesterol. The transcription factor (TF) binding site enrichment analysis of the promoter region of *CYP94D144* revealed NAC TF as a potential regulator. The results were further validated through expression profiling by qRT-PCR, and the comparison of high and low diosgenin producing hybrids obtained from a bi-parental population.

**Conclusions:**

This study not only enhanced the understanding of the genetic basis of diosgenin biosynthesis but also serves as a valuable reference for future genomic investigations on *CYP94D144*, with the aim of augmenting diosgenin production in yam tubers.

**Supplementary Information:**

The online version contains supplementary material available at 10.1186/s12870-024-05133-1.

## Background

Diosgenin stands as a pivotal steroidal precursor [1] widely employed in initiating the synthesis of various compounds including androgens, antioxidants, oestrogens [2], and contraceptives [3–8]. Its potential in anticancer therapy has drawn significant attention from medicinal and synthetic chemists [9]. Various bioactivities of diosgenin as anti-tumor [10], and bone-protecting [11] properties have been reported [12]. In recent years, it has emerged as an efficient and increasingly sought-after oral contraceptive [12]. Due to its importance and involvement in the production of more than 300 types of steroid hormones, including anabolic hormones, corticosteroids, sexual hormones, and proteins, the annual market demand for diosgenin continues to surge at an approximate rate of 8% [13, 14]. Consequently, there is an imperative need to explore new genetic resources and enhance existing germplasms to meet the escalating demand for high-quality diosgenin materials.

The *Dioscorea* species are well-known for their diosgenin content. There are over 600 reported *Dioscorea* species, of which 41 contain more than 1% diosgenin in their tubers [15]. The booming market demands leads to the worldwide cultivation of *Dioscorea* species. *Dioscorea zingiberensis* C. H. Wright commonly known as “yellow ginger” holds significance as an essential medicinal herb and a crucial genetic resource of diosgenin in China. It is widely distributed in the southern China from Hubei to Shaanxi, Hunan, Sichuan provinces [16]. China ranks among the top diosgenin-producing countries [17]. *Dioscorea zingiberensis* is well-recognized in Traditional Chinese Medicine for its efficacy in treating various ailments, including anthrax, cough, sprains, arthritis, and cardiac diseases [18, 19]. *Notably, it can accumulate diosgenin ranging from 2 to 16% in its rhizomes* [20]. While various extraction processes of diosgenin from D. zingiberensis and its industrial applications have been studied, the lack of high-quality germplasm and genomic information for diosgenin production hampers overall yield and synthetic biosynthesis efforts. The complete list of genes involved in diosgenin biosynthesis are still not available. A transcriptome study revealed the differentially expressed genes in rhizomes of high and low diosgenin producing plants [8]. Previously, the labeling-based studies have suggested the cholesterol as a precursor of diosgenin biosynthesis [21–23]. In plants, the genes contributing to the conversion of cycloartenol to cholesterol have been identified [24]. Cholesterol can be transformed into diosgenin through oxidative modifications at the C-16, C-22, and C-26 positions by P450-dependent enzymes such as, dioxygenases, monooxygenases and other catalysts, followed by the addition of rhamnose and glucose to the C-3 position of diosgenin molecules by UDP- glycosyltransferases (UGTs) [12]. The specific steps in diosgenin biosynthesis have previously been proposed [25], but the genes associated to these steps have not been reported [12].

Genetic mapping and gene manipulation of metabolic pathways represent promising strategies for developing varieties of pharmaceutically important plants with enhanced medicinal compounds. Nonetheless, only a few genomic regions (QTLs/genes) associated with diosgenin biosynthesis have been reported and cloned [26]. Conventional gene mapping procedures are expensive, time-consuming, and provide a limited genetic information, which limits the understanding of diosgenin biosynthesis pathway [8]. The development of Genotyping-By-Sequencing (GBS) technology [27] and the advancements in bioinformatics tools have opened new opportunities to unveil the genetic background and genomic regions associated to diosgenin production. GBS is a high-throughput genotyping method that simultaneously identifies and scores genetic variations across the genome. GBS involves sequencing a reduced representation of the genome, typically achieved through restriction enzyme digestion followed by sequencing of the resulting fragments [27]. The genome-wide association study (GWAS) is a method used to identify genetic variations associated with a particular trait or disease in a population. It scans the entire genome of individuals to pinpoint genetic variants that are more common in individuals with the trait of interest compared to those without it. Taking advantage from GBS, GWAS has been implemented to detect the QTLs and candidate genes controlling important traits in plants [28]. No GWAS has been reported in *D. zingiberensis* and no genetic study has been conducted on diosgenin variation.

To identify the QTLs and candidate genes associated with diosgenin variation in *D. zingiberensis* tuber, we performed a GWAS. It enabled us to locate the major genetic variants in the *D. zingiberensis* genome controlling diosgenin content. Additionally, we established a bi-parental population, segregating into high and low diosgenin-producing pools, to validate the results obtained from GWAS. This study will pave a way for the development of *D. zingiberensis* cultivars with enhanced diosgenin content.

## Methods

### Plant materials and experimental conditions

A diverse population of 180 accessions was designed to find the genomic regions associated with the diosgenin contents in *D. zingiberensis* (Supplementary Table 1). The accessions were originally collected from three provinces located in the southwestern part of China (Yunnan, Sichuan, and Guangxi) but no detailed passport data are available. The plant materials were formally identified by Prof Yunpeng Luan and all germplasms are conserved as vitroplant at the Genebank of Southwest Forestry University. No permission is required to work on this species. The genotypes were planted at two different agro-ecological conditions at Luohe in 2021 (33^o^ 34’ 18’’ North and 114 ^o^ 2’ 7’’ East) and Hainan in 2022 (18^o^ 56’ 22’’ North and 109 ^o^ 29’ 3’’ East), two cities of China. Luohe and Hainan exhibit distinct differences in climate, soil types, and vegetation due to their geographical locations within China. While Luohe has a temperate continental climate and is dominated by agricultural landscapes, Hainan experiences a tropical climate and boasts diverse tropical vegetation, including rainforests and mangroves. Hainan Island, located in the southern part of China, has a tropical climate influenced by its proximity to the equator and the surrounding ocean. It experiences high temperatures year-round, with average temperatures ranging from 24 °C to 28 °C. The climate is characterized by high humidity and abundant rainfall, particularly during the wet season from May to October. Hainan Island has a diverse range of soil types, including red soil, laterite soil, and tropical forest soil. Luohe, located in Henan Province, is situated in the central part of China. It typically experiences a temperate continental climate with distinct seasons. Summers are hot and humid, with temperatures often exceeding 30 °C, while winters are cold and dry, with temperatures dropping below freezing. Spring and autumn are relatively mild. The predominant soil types in the Luohe area are those associated with the North China Plain, such as various types of loam, silt, and clay soils. These soils are fertile and suitable for agriculture.

Due to the significant intra-varietal variability in yam, it is crucial to plant multiple tuber fragments. We planted 5 tuber fragments per variety, resulting in 5 plants. To address spatial heterogeneity, we distributed the planting across 3 distinct ridges according to the randomized complete block design. Therefore, each accession was represented by a total of 15 plants (5 plants * 3 ridges). All the standard cultural practices were kept constant as per local requirements [29]. The plants were harvested at the time of senescence (ranging from 9 to 10 months).

### Sample preparation and evaluation of diosgenin content

The fresh tubers obtained from *D. zingiberensis* plants harvested in November-January were peeled and used to determine the diosgenin contents. For each accession, slices (~ 3 cm thickness per tuber) from the middle of 3 tubers randomly selected per ridge (biological replicate) were finely cut and mixed. It is worth noting that a single plant can yield up to 10 tubers. We collected 1 g of air-dried samples, placed them in twice volume of distilled water in a conical flask. These were further hydrolyzed with 50 ml of 2.0 N H_2_SO_4_ at 1.15 Pa pressure and 100 °C temperature for 6 h in a pressure cooker. The contents were then cooled and filtered by using Whatman filter paper No. 1. To achieve a neutral pH (7.0), the filtered residue was then washed with distilled water. The neutralized and filtered residue was dried and then extracted with 50 ml petroleum ether for 6 h at 80 °C using the Soxhlet extraction method [20]. The extract was dried in rotary evaporator and the recrystallization was performed using ethanol as solvent [20, 30]. The final crystal was re-dissolved in 25 ml ethanol for high performance liquid chromatography (HPLC) analysis. The chromatography was performed using the MeOH: H_2_O (95:5; v/v) as elution solvent at 0.5 ml min^−1^ flow rate, Econobase C18 column was 4.6 mm × 150 mm, 5 μm. The absorbance was recorded at wavelength of 203 nm.

### Statistical analysis for phenotypes

The R-program was used to estimate and visualize the frequency distribution, descriptive statistical, and Q-Q plots for diosgenin contents. The significance of available phenotypic diversity in the population and across locations was also estimated by analysis of variance in R-program.

Variance estimates were used to estimate broad–sense heritability (*h*^*2*^) according to the formula: *h*^*2*^ = σ^2^g/(σ^2^g + σ^2^ge + σ^2^e), where σ^2^g, σ^2^ge and σ^2^e are the variance components for genotypes, genotypes × location_replicate and residual variation, respectively.

The best linear unbiased prediction (BLUP) was estimated with the package lme4 (R.2.15) with the following model:

Phenotype~(1|Genotype) + (1|Location) + (1|REP%in%Location) + (1| Genotype: Location).

### Genotyping and population clustering analysis

The fresh leaf samples were collected from different plants (3-month-old) of each accession and mixed. The whole genome DNA was extracted by extraction kit (Imagene, China) as per manufacturer’s protocol. The quality of the DNA was checked using a Nanodrop 8000 spectrophotometer (Thermo Fisher Scientific, Waltham, MA, USA). All of the DNA samples were subject to GBS a 96-plex Pst I GBS protocol [31]. Briefly, the DNA of each accession was digested with the restriction enzyme PstI (New England Biolabs, Beijing, China). Restriction cutting sites were ligated with adapters (barcodes) with the T4 ligase. The ligated products were then pooled together. Single-end sequencing was performed using an Illumina HiSeq2500 instrument (Illumina Inc. San Diego, CA, USA). The generated raw reads were processed (sorting, demultiplexing and trimming) using the TASSEL-GBS v2 pipeline [32]. The mapping onto the reference genome [33] was performed using the Burrows–Wheeler alignment (BWA) v0.7.17, and the SNPs were called with DiscoverySNPCallerPluginV2. The average sequencing-error-rate per base was set to 0.01, while the threshold quality score value for a marker position was set to zero. The missing data was determined by TASSEL 5.0 software [34] and the minimum count was set to 75%. The SNPs with the minor allele frequency (MAF) less than 0.05 were filtered. The population relatedness (kinship, k-matrix) based on the VanRaden method [35] was conducted in the GAPIT program (http://www.maizegenetics.net/GAPIT) [36] while the principal component analysis (PCA) was performed based on FactoMineR package [37].

### Population structure analysis

The population genetic structure of the 180 accessions was inferred by using a Bayesian model-based method in STRUCTURE v2.3.4. The number of population clusters was predetermined as k ranging from 1 to 10. We applied five independent runs for each k. Each run involved a total of 100,000 Markov chain Monte Carlo iterations after a burn-in period of 100,000 iterations. We determined the best k population following the Evanno ΔK method [38].

### Genome wide association analysis

The top 10,000 high quality SNPs were saved in vcf file format. The genotypic data along with diosgenin contents were used to perform the genome wide association study (GWAS) in GAPIT [36]. The mixed linear model (MLM) with kinship matrix was used for marker trait association using the following equation:

$$y=G\beta +P\mu +\text{K}+\text{e}$$

where *y* was the vector of observations, *β* and *µ* were vectors of fixed and random effects, respectively, *G* denoted the genotypic (SNP markers) matrix, *P* was the phenotypic data matrix, the kinship matrix (*K*) was used as covariate, and *e* was a random residual vector. The BLUP values [37] were used to identify the loci controlling the diosgenin content. It is worth noting that recent GWAS models such as FarmCPU and BLINK were also tested, producing results identical to those obtained with the MLM. Consequently, only the results from the MLM are presented in this manuscript.

The significant association and QTL regions were defined at a threshold of –log10 (P) ≥ 5 (0.05/n, *n* = 10,000 SNPs). The significant SNPs locations were searched in the genome GFF file to find out the candidate genes. To predict the potential transcription factors controlling the candidate gene, the Plant Transcriptional Regulatory Map (PlantRegMap) platform (http://plantregmap.gao-lab.org/tf_enrichment.php) was used.

### External validation of the candidate QTL

The identified loci which were significantly associated with diosgenin contents were further validated by comparing the low and high pools of genotypes in a separate bi-parental population. Parent A (G0XT12) characterized with 2.62% diosgenin content was crossed to Parent B (G0XT422) known for high (13.55%) diosgenin content. Five hybrids with low diosgenin content (< 3%) were compared with five hybrids containing high diosgenin content (> 10%). DNA were extracted from the leaf samples using the CTAB method. With the SnapGene 6.1 software (www.snapgene.com), we designed PCR primers (5’-TGAGGGGTTTCTGGGAGG-3’; 5’-ATAGGTGTTGAGTTGGCGG-3’) to amplify 300 bp in the promoter region of the candidate gene around the peak SNP. PCR sequences were aligned in MEGA10 software [39]. Next, the total RNA was extracted from tubers and expression pattern of the candidate gene was further evaluated by qRT-PCR in both high and low hybrid pools based on previous reported descriptions [40]. Briefly, the qRT-PCR experiment was conducted in an Applied Biosystems™ 7500 Real-Time PCR machine (Thermo Fisher Scientific, Waltham, Massachusetts, USA) with a SYBR Green PCR Master Mix (Tiangen Biotech, Beijing, China). Total RNA was extracted with RNAprep Pure Plant Kit (Tiangen Biotech, Beijing, China), and the RNA was transcribed with Quantscript Reverse Transcriptase Kit (Tiangen Biotech, Beijing, China). We pooled equal volumes of cDNA from each male individual into one tube and equal volumes of cDNA from each female individual into another tube. This creates separate male and female cDNA pools. A primer pair (5’- TGCAAAACTCACCAGGTTCA − 3’; 5’- AAGGATGAGCTTATGCGGAA − 3’) was designed using PrimerPremier5. The relative expression of the candidate gene was quantified following the comparative CT method [41]. Three technical and three biological replicates were applied, and the expression data were normalized against *D. zingiberensis* actin gene sequence (NCBI GenBank accession: JN693499).

## Results

### Diversity and heritability for diosgenin in *D. zingiberensis*

**Fig. 1:**
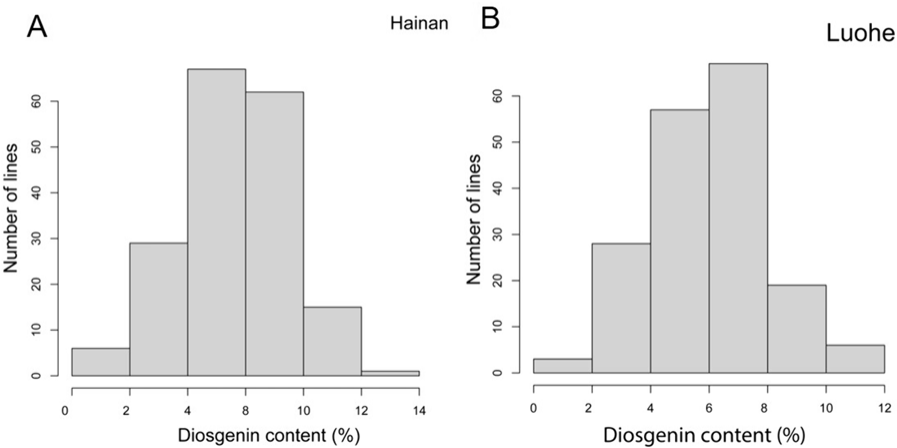
Variation of diosgenin content among the association panel at two planting sites (**A**) Hainan and (**B**) Luohe

A diverse population of 180 selected *D. zingiberensis* genotypes was evaluated for the diosgenin contents. The extent of variation among the genotypes was estimated in two environmental conditions at Hainan and Luohe (China). A significant variability among genotypes was observed for diosgenin contents at both locations (Table 1). A consistent normal frequency distribution was observed for diosgenin contents at both locations (Fig. 1; Table 1). It showed that diosgenin content is a typical quantitative trait that is governed by the contribution of several genes. Moreover, the phenotypic data is suitable for genome wide association study (GWAS). We observed a relatively higher average value of diosgenin contents at Luohe while a wider range was observed at Hainan (Table 1). A genetic and environmental variations among the genotypes were observed by the analysis of variance and coefficient of variation (Table 1). The genotypic variations along with significant broad sense heritability (*h*^*2*^) demonstrated the genotypes as the main source of variation for diosgenin contents in the population which could be inherited to next generations. There was no effect of environmental factors on diosgenin production. However, the genotype by environment interaction was significant.

**Table 1:** Descriptive statistics for diosgenin content in the GWAS panel

Parameters	Luohe	Hainan
Mean (% contents)	4.7	4.4
Min (% contents)	1.23	1.12
Max (% contents)	11.55	13.31
Coefficient of variation (%)	64.59	49.12
Heritability (*h*^*2*^)	0.86	
Genotypic variance	***	
Environmental variance	ns	
Genotype to Environment interaction	**	

***significance *P* <0.001, **significance *P* <0.01

*ns* non-significant

### Genotyping and population structure analysis

**Fig. 2:**
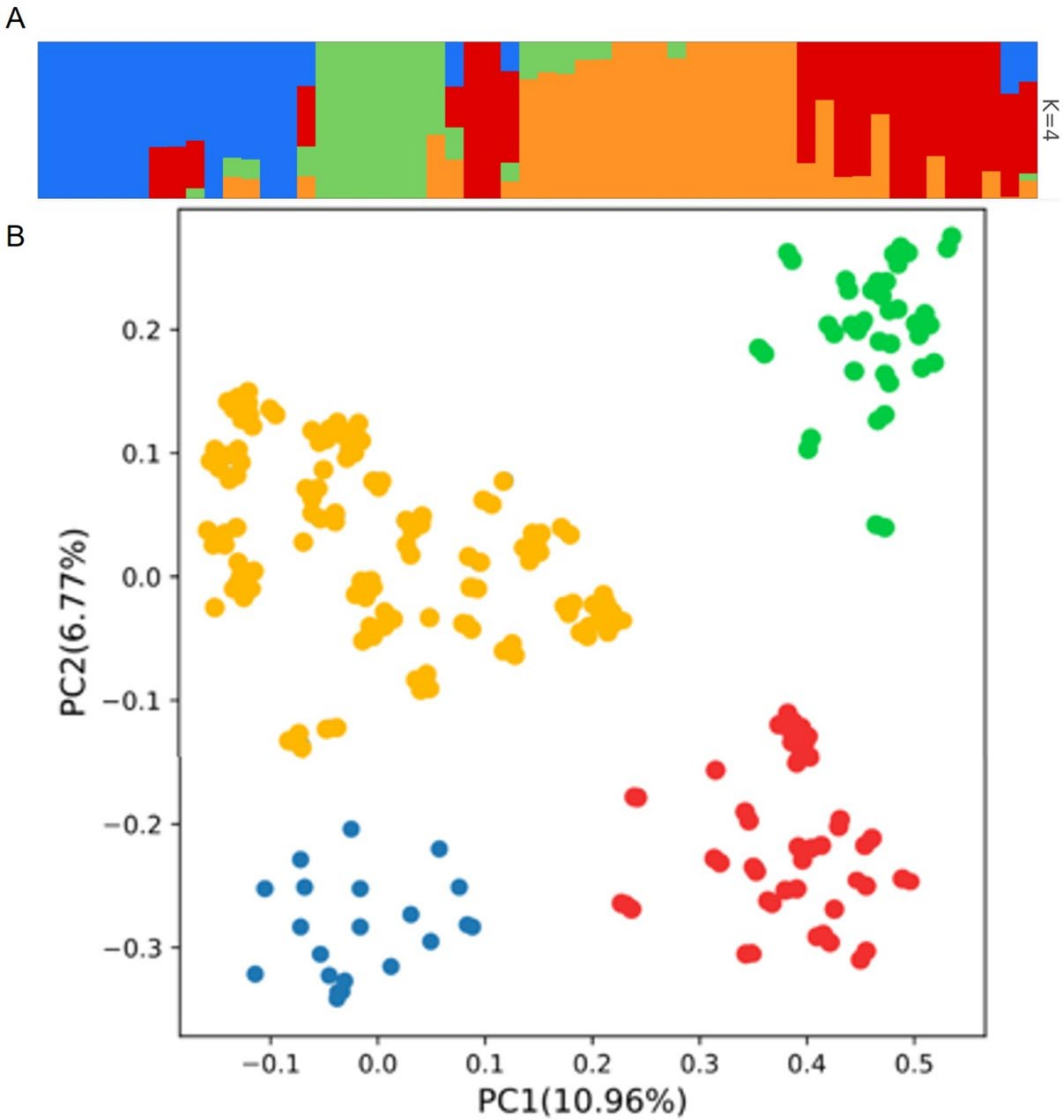
Population structure (**A**) and principal component (**B**) analyses of the *D. zingiberensis* accessions

 The genotyping-by-sequencing approach was used to genotype the whole population, and the top 10,000 SNP markers were obtained after strict screening criteria. The SNPs were well-distributed in the *D. zingiberensis* genome with a marker density of 9.6 SNPs/kb. The population structure and principal component analysis (PCA) were conducted. Both PCA and Structure analysis showed four (K = 4) main groups genotypes (Fig. 2A, B). The PC1 only explained 10.96% of the total variation, confirming that the diversity is so broad that many components are required to explain it.

### Genome wide association study and the mining the candidate genomic region

**Fig. 3:**
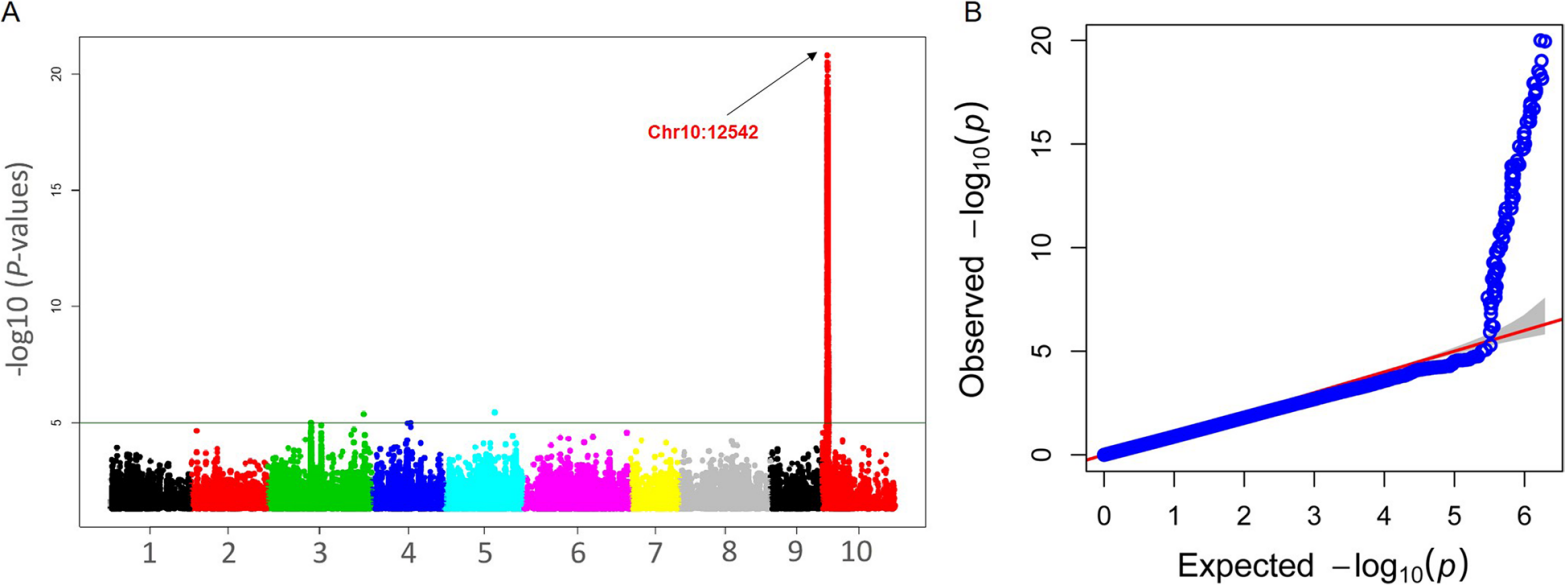
Genome-wide association mapping for diosgenin content in *D. zingiberensis*. Manhattan plot for diosgenin content (**A**). Quantile-quantile plot for diosgenin content (**B**)

 The GWAS was performed to identify the genomic regions associated with diosgenin production in *D. zingiberensis*. The mixed linear model was adopted for the regression analysis with population structure data as covariate. The threshold –log10 (p) ≥ 5 was considered for the identification of significant marker-trait associations. A total, 93 SNPs mainly on chromosome 10 were observed to be significantly associated with the target trait (Fig. 3A, Supplementary Table 2). All the associated SNPs were clustered in a 1,150 bp genomic region. Hence, we defined this region as a QTL. The peak SNP at the 12,542 bp position on physical map of chromosome 10 showed a very strong association (–log10(p) = 20.63) with diosgenin production. The SNP could explain 64% of the diosgenin variation in the *D. zingiberensis* tuber, showing that it is a major QTL. The deviation from expected values of SNP markers was estimated by Q-Q plot by GAPIT in R-program, and a significant deviation of SNPs was observed (Fig. 3B).

### Detecting genes in the QTL region

**Fig. 4:**
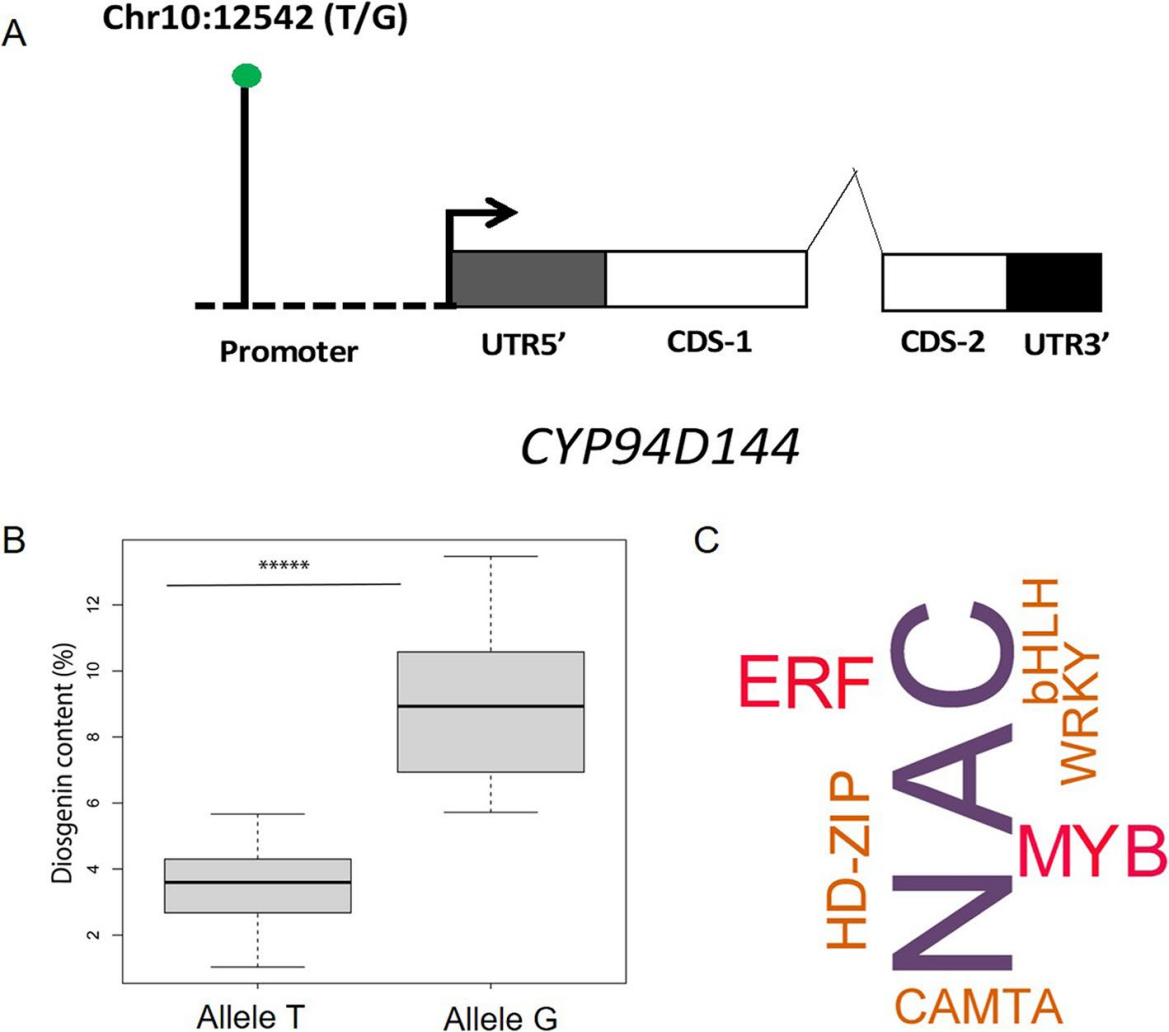
Characterization of the structure of D. zingiberensis CYP94D144 gene showing the location of the SNP Chr10:12542 (T/G) in the promoter region (**A**). Comparative quantification of diosgenin content for accessions exhibiting T and G alleles (**B**). Analysis of the transcription binding factors in the promoter region of CYP94D144 gene (**C**). *** means significant difference at *p *< 0.001

 Based on the genome GFF file, we located the peak SNP at 202 bp distance within the promoter region of the candidate gene annotated as *CYP94D144* (Fig. 4A). The top associated SNP markers had two alleles (G/T) (Fig. 4B), hence, the allelic performance was estimated. The allele G was observed to be responsible for the higher diosgenin contents in *D. zingiberensis* tuber. We speculated that the peak SNP alters the binding of a regulator gene (such as a transcription factor (TF)) controlling the expression of *CYP94D144*. To predict the potential TF controlling *CYP94D144*, the Plant Transcriptional Regulatory Map (PlantRegMap) platform (http://plantregmap.gao-lab.org/tf_enrichment.php) was used for *in-silico* TF binding site enrichment analysis in the promoter region of *CYP94D144*. Seven TF families were predicted but the NAC TF binding domain was the more enriched, hence could be involved in the regulation of *CYP94D144* (Fig. 4C).

### Validation of GWAS results in an external panel

**Fig. 5:**
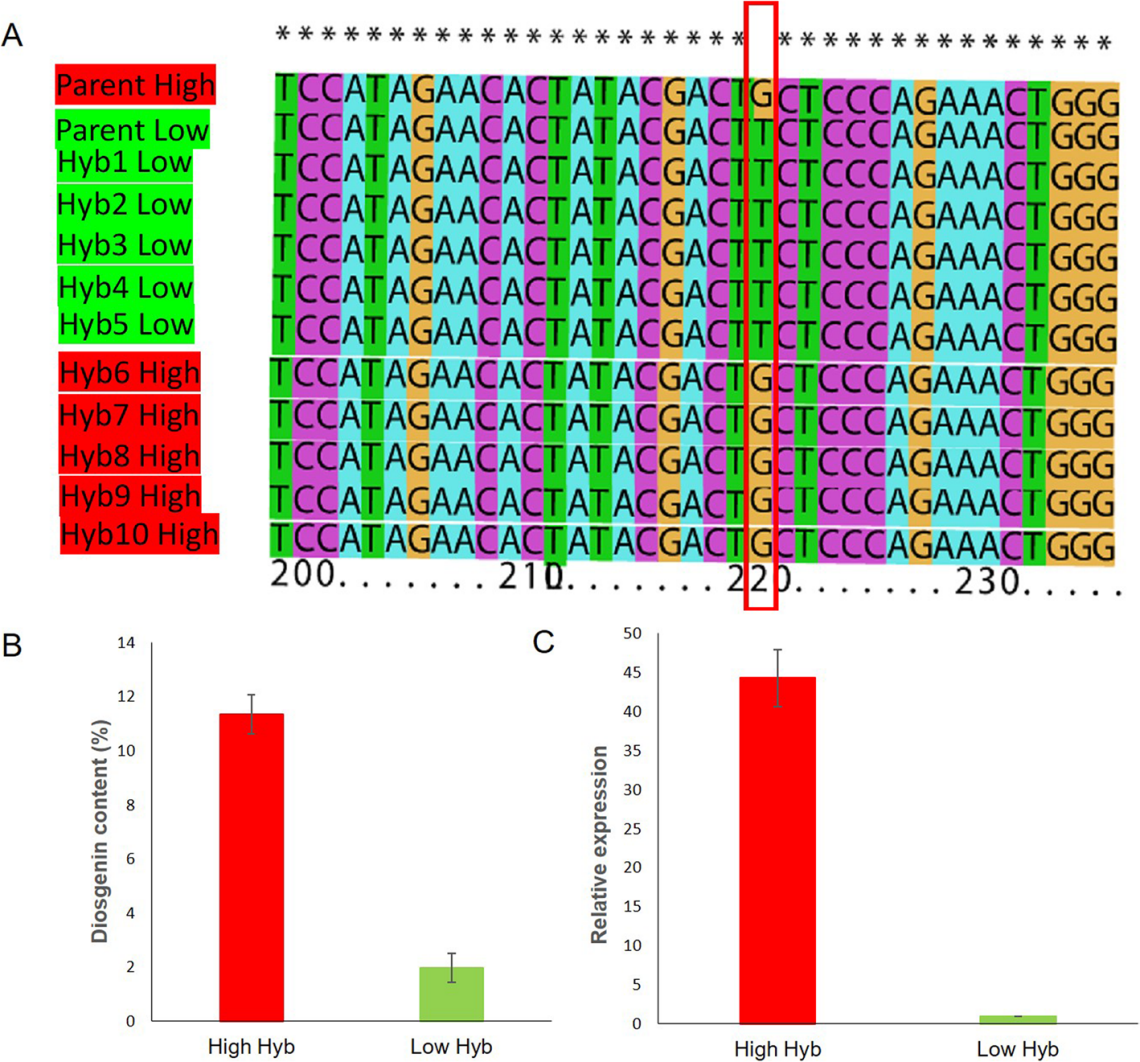
Validation of the SNP Chr10:12542 in contrasting hybrids derived from a mapping population (**A**). Comparative quantification of diosgenin content in two contrasting pools of hybrids for SNP alleles and diosgenin content (**B**). Relative expression of the both versions of the gene via qRT-PCR experiment (**C**)

 To validate the detected marker trait association, an external panel of bi-parental hybrids (a group of five hybrids plants with low diosgenin content and a group of five hybrids plants with high diosgenin content) was used (Fig. 5A). The sequencing PCR product (300 bp promoter region of *CYP94D144*) and alignment showed that both parents possess different alleles at the identified peak SNP through GWAS. Similarly, all hybrids with low and high diosgenin content possess the corresponding alleles (Fig. 5A). Individuals with high diosgenin content harbored the G allele of, while individuals with the T allele had low diosgenin content (Fig. 5B). The qRT-PCR expression profiling of *CYP94D144* in the two pools was conducted. A very low expression level (40X lower) of *CYP94D144* was observed in the pool of hybrids with the T allele as compared to the pool of hybrids harboring the G allele (Fig. 5C). This result indicates that a natural variation in the promoter region of *CYP94D144* potentially alters the binding of a regulatory gene leading to its differential expression and impacting the biosynthesis of diosgenin in the tuber.

## Discussion

### Phenotypic assessment of association panel

Diosgenin, a steroidal sapogenin, serves as a fundamental precursor in the production of various steroidal medicines, underpinning its significant pharmaceutical relevance. The *Dioscorea* species are the main sources of diosgenin [42]. In yam, a recent study identified and validated various genes linked to diosgenin biosynthesis [43]. In this study, we used a diverse panel of *D. zingiberensis* to evaluate the diosgenin contents. The range of diosgenin production was wider in Hainan than Luohe, which is due to significant genotype by environmental interactions (Table 1). A similar effect has also been reported for starch production in other crops like Tobacco [44]. However, the genotypes with high diosgenin contents in Hainan also showed high contents in Luohe. It revealed that the genetic differences were the main source of variation. The 86% heritability also supported the genotypic effect in the population. Hence, it can be concluded that diosgenin content variation is mainly governed by genetic factors. The wider phenotypic range and normal frequency distribution of genotypes in population suggest the suitability of this population for GWAS to evaluate the genomic associations with diosgenin contents.

### Population structure assessment of association panel

GWAS is a powerful approach to reveal the genomic regions associated with complex quantitative traits. However, the relatedness among genotypes caused by population structure may result in false positive identifications [44]. In this study, the SNP marker-based population structure revealed four sub-populations by PCA and K-matrix. Unfortunately, there is no information on the geographical origins or breeding status of the accessions to clarify the underlying patterns of the clustered sub-populations. The Type-I errors (false positives) in GWAS were controlled by involvement of covariates based on population structure, kinship matrix, and the principal components. The same strategy has been adopted by various researchers for precise GWAS evaluation [28, 44, 45].

### Genome wide association study for diosgenin contents

The diosgenin biosynthesis in *D. zingiberensis* is a complex trait [42, 43, 46]. Based on transcriptome study, various genes involved in diosgenin metabolic pathways have been reported [43]. In this study, we performed the GWAS using the mix linear model and estimated the BLUP values. Hence, we found 93 SNP markers over the threshold. All of these SNPs were clustered on chromosome 10 with well-fitted Q-Q plot. Similar results have been reported on starch contents in potato [47]. The clustering of most significant SNPs at a single locus on chromosome 10 indicates the availability of a single locus with a major contribution to diosgenin biosynthesis. Nonetheless, we believe that many minor effect loci controlling diosgenin content were not detected in this study. Two key explanations for this result: (1) the size or diversity of the population is not enough to detect these loci; (2) The SNP density from the GBS approach is low. Dossa et al. [48] recently demonstrated that a high marker density is crucial for genuinely deciphering the genetic architecture of complex traits in yams.

### Candidate gene regulating the diosgenin biosynthesis

The expression analysis and/or allelic performance have been widely reported to understand the molecular function of candidate genes and specific loci [44]. It has been reported that the allelic polymorphisms within the gene region or in promoter region are able to control the gene expression pattern [49]. Hence, the expression analysis for candidate gene (*CYP94D144*) was performed and the results were validated. The allelic performance of significantly associated marker was also tested and validated by two-tailed paired Students’ T-test. These speculate the candidate gene (*CYP94D144*) as a major contributor in diosgenin biosynthesis pathway.

The candidate gene belongs to the P450 gene family. In a previous study, it has been discovered that the development of CpG islands control the carbon flux between starch and diosgenin production [43]. This is a result of duplication and neo-functionalization of P450 genes in diosgenin biosynthesis pathways [42, 43]. The diosgenin biosynthesis is a result of continuous metabolic-oxidative reactions on steroidal skeleton compound cholesterol at C-16, C-22, and C-26 [50]. The cholesterol molecules hydroxylation is known to be catalyzed by cytochrome P450 (CYP) enzymes [51].

While *Dioscorea* species yield a significant amount of diosgenin, the mechanisms governing its biosynthesis, emergence, or evolution in plants remain unexplored [42]. . Various metabolic engineering approaches have been employed to understand and improve the production of diosgenin in yeast [52]. The diosgenin biosynthetic pathway has evolved from the modification of the competing starch-biosynthesis pathway [43, 51]. Many CYP related genes in these pathways were revealed by transcriptome analysis of *D. zingiberensis* [8, 51]. However, no study has been designed to investigate the genetic variants associated with variation of diosgenin content in *D. zingiberensis* tuber.

The CYP genes are categorized into subfamilies of P450 (i.e., CYP71D55, CYP75A, CYP76, CYP77A, CYP78A5, CYP93E, CYP701, CYP707, CYP716A, CYP73A, CYP74A), which are known to contribute to various metabolic pathways, including fatty acid, flavonoid, indole alkaloid, gibberellin, Abscisic acid (ABA) and sesquiterpenoid, metabolisms [51]. The CYP-encoding genes are reported for diosgenin biosynthesis [51]. Previously, a total 485 CYP encoding genes were annotated in *D. zingiberensis* genome as potential candidates for diosgenin biosynthesis and accumulation [51]. The *CYP94D144* is an ortholog of two P450 genes *i.e., CYP90G4* in *Paris polyphylla* and *CYP90B50* in *Trigonella foenum–graecum* [43]. These genes are involved in the independent biosynthesis of diosgenin in *Dioscorea* from cholesterol corresponding to the C-26 hydroxylase steroids, and C-16, 22-dihydroxylase [43, 53]. Cholesterol is a precursor molecule for diosgenin biosynthesis [8, 50, 51, 53]. The steroidal saponins could be biosynthesized from C5 units, isopentenyl diphosphate (IPP). These IPP may derived either from the plastidic methylerythritol phosphate pathway or the cytosolic mevalonate pathway. The *CYP94D144* along with *CYP90B71*, and *CYP90G6* constitute a gene cluster that governs the diosgenin biosynthesis and has been commonly reported in diosgenin producing plant species [43]. Hence, we can conclude that the *CYP94D144* along with other P450 genes (*CYP90B71*, and *CYP90G6*) play a major role in diosgenin biosynthesis in *D. zingiberensis*. In another experiment, *CYP94D144* was expressed in the cholesterol-producing-yeast (DG-Cho) [43, 52]. It resulted in a new yeast strain (DG002) that can successfully convert the cholesterol to diosgenin. A deeper *in-vivo* investigation on *D. zingiberensis* by gene knockout experiment is required to reveal the role of *CYP94D144*.

## Conclusions

The current study revealed the gene *CYP94D144* as a major contributor of diosgenin content in *D. zingiberensis.* The identification of the associated marker and the allele responsible for high diosgenin content will be useful for future breeding projects aiming at developing materials with increased diosgenin content. This study can be a reference to isolate and to further characterize the gene *CYP94D144* for deeper insights into its function. It will open new horizons of ectopic diosgenin biosynthesis in order to satisfy its high demand for medicinal purposes.

### Supplementary Information

Supplementary Material 1.

Supplementary Material 2.

## Data Availability

The raw sequencing data are available at NCBI SRA under the project number: 716093 (https://www.ncbi.nlm.nih.gov/bioproject/716093).
